# A protocol for *Chenopodium quinoa* pollen germination

**DOI:** 10.1186/s13007-022-00900-3

**Published:** 2022-05-18

**Authors:** S. Elizabeth Castillo, Jose C. Tovar, Anastasia Shamin, Jorge Gutirerrez, Paige Pearson, Malia A. Gehan

**Affiliations:** Donald Danforth Plant Science Center, St. Louis, MO 63132 USA

**Keywords:** Quinoa, Pollen germination, Protocol

## Abstract

**Background:**

Quinoa is an increasingly popular seed crop frequently studied for its tolerance to various abiotic stresses as well as its susceptibility to heat. Estimations of quinoa pollen viability through staining methods have resulted in conflicting results. A more effective alternative to stains is to estimate pollen viability through in vitro germination. Here we report a method for in vitro quinoa pollen germination that could be used to understand the impact of various stresses on quinoa fertility and therefore seed yield or to identify male-sterile lines for breeding.

**Results:**

A semi-automated method to count germinating pollen was developed in PlantCV, which can be widely used by the community. Pollen collected on day 4 after first anthesis at zeitgeber time 5 was optimum for pollen germination with an average germination of 68% for accession QQ74 (PI 614886). The optimal length of pollen incubation was found to be 48 h, because it maximizes germination rates while minimizing contamination. The pollen germination medium’s pH, boric acid, and sucrose concentrations were optimized. The highest germination rates were obtained with 16% sucrose, 0.03% boric acid, 0.007% calcium nitrate, and pH 5.5. This medium was tested on quinoa accessions QQ74, and cherry vanilla with 68%, and 64% germination efficiencies, respectively.

**Conclusions:**

We provide an in vitro pollen germination method for quinoa with average germination rates of 64 and 68% on the two accessions tested. This method is a valuable tool to estimate pollen viability in quinoa, and to test how stress affects quinoa fertility. We also developed an image analysis tool to semi-automate the process of counting germinating pollen. Quinoa produces many new flowers during most of its panicle development period, leading to significant variation in pollen maturity and viability between different flowers of the same panicle. Therefore, collecting pollen at 4 days after first anthesis is very important to collect more uniformly developed pollen and to obtain high germination rates.

**Supplementary Information:**

The online version contains supplementary material available at 10.1186/s13007-022-00900-3.

## Introduction

Quinoa (*Chenopodium quinoa* Willd) has grown in popularity due to its ability to produce highly nutritious grains in low quality soils [1–6]. To expand quinoa cultivation to new geographical areas and to develop new varieties, more research to understand quinoa responses to the environment is needed. Since quinoa is a seed crop, one key aspect of quinoa research is determining how different environments and stresses affect pollen viability [7–10]. Pollen viability is also very helpful to establish male sterility, which greatly facilitates plant breeding and variety development [11, 12].

Pollen viability can be directly quantified through microscopy of pollen tubes forming in styles [7, 13], but this is a complicated and labor-intensive method. A common method of estimating pollen viability is through various pollen stains. However, pollen staining methods can overestimate the proportion of pollen that is physiologically viable [14, 15], and different staining methods can produce inaccurate results [14–16]. In quinoa, Alexander pollen staining showed no significant changes in pollen viability under heat stress [8], while tetrazolium pollen staining showed a significantly lower pollen viability under a similar heat stress treatment [9]. In vitro pollen germination assays are more accurate than staining methods and much simpler than direct quantification of pollen tubes forming inside styles [9, 13, 17].

Pollen germination assays determine the viability of a pollen grain by its ability to form a pollen tube in vitro. In vitro pollen germination media requires three essential components: sugar, boron, and calcium nitrate [16, 18]. Sucrose acts as an energy source and maintains osmotic balance [18]. Boric acid facilitates growth of the pollen tube, membrane permeability, structure and sugar transportation, while calcium controls the permeability of the pollen tube membrane and assists in ion balance [16, 18].

Here, we report a new method for in vitro pollen germination of quinoa. This protocol was tested on two quinoa accessions that were selected because they represent different geographic origins and are genome sequenced. QQ74 (PI 614886) is a coastal Chilean accession, and cherry vanilla is a variety developed in Oregon [19]. We modified a pre-established medium for in vitro pollen germination of the closely related sugarbeet (*Beta vulgaris* L) [16] to obtain an efficient quinoa pollen germination medium. We also study how flower development and pollen incubation time influence pollen germination in vitro to determine the optimum time for pollen collection and incubation in pollen germination media. We also developed a semi-automated python-based bioinformatics tool to count germinated pollen from image data in PlantCV [20, 21]. Together, this protocol and this image analysis tool represent an important protocol for the quinoa community and a generalizable tool that can speed the process of assessing pollen germination.

## Results

### Development of a semi-automated method in plantCV to count pollen

**Fig. 1:**
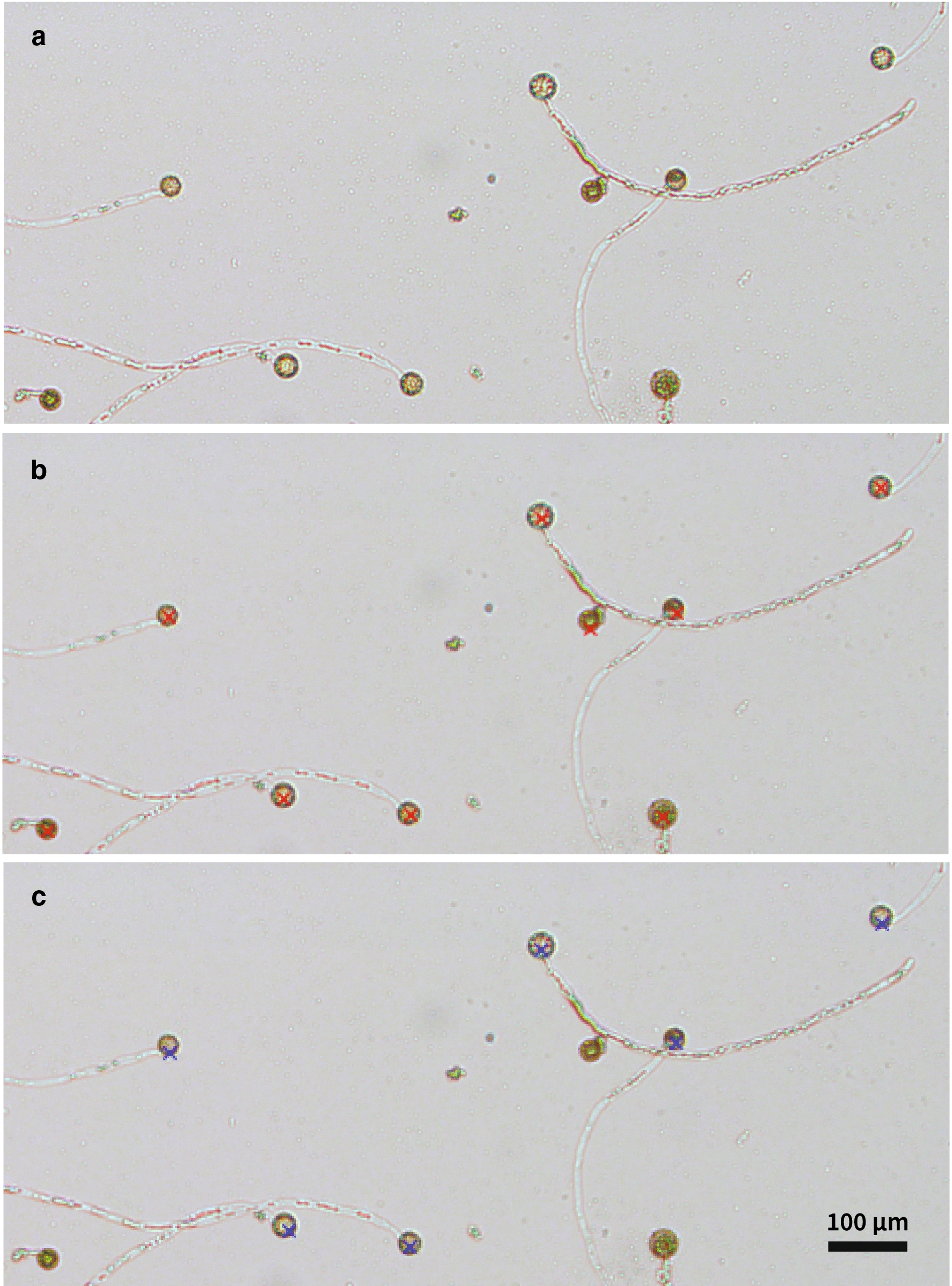
**a** Close-up of a representative image of germinated and ungerminated pollen grains from quinoa QQ74. **b** The same close-up image showing all automatically and manually labeled pollen grains in PlantCV marked with red Xs. **c** The same close-up image showing only the manually labeled germinated pollen grains in PlantCV marked with blue Xs

To reduce the time spent manually counting the pollen grains and germinating pollen tubes in image data (Fig. 1a), we developed a PlantCV workflow that semi-automates the process of detecting pollen grains. The workflow consists of two main steps, first segmenting the objects (pollen grains and tubes) from the background, and then identifying only the objects that are likely to be pollen grains. After these two automatic steps, the user is required to manually correct wrong detections and select missing grains (Fig. 1b).

In order to detect the pollen grains, we measured the eccentricity of each connected region in the segmentation. This was done using the new PlantCV function ‘*plantcv.detect_discs*’ that was developed to help automate pollen detection*.* Circular objects have values of eccentricity close to 0. Thus, the closer the eccentricity value is set to 0 in the PlantCV function ‘*plantcv.detect_discs*’, there will be fewer false positives, but also less pollen grains will be automatically detected. Although this semi-automated tool relies on the user to perform visual inspection and manual correction of the automated detection within the tool, it is faster than alternative fully manual tools such as ImageJ. We identified pollen grains in the images by detecting any object with eccentricity under a value of 0.65.

We then imported the results in the new PlantCV tool ‘*plantcv.visualize.ClickCount’* where users can manually refine the detections. For example, users can add any pollen grains that were not automatically detected or remove any false positive pollen. We used this same tool to manually identify the germinated pollen (Fig. 1c). In the end we obtain the total number of pollen grains (Fig. 1b) and the number of germinated pollen grains (Fig. 1c). From these measurements we can determine the percent of germinating/total pollen grains. Although this open-source tool was developed for counting quinoa pollen, these image analysis tools and methods are likely generalizable to images of circular pollen from other species and for other applications. For example, automated detection of round seeds in image data.

### Optimization of sucrose concentration in pollen germination media

**Fig. 2:**
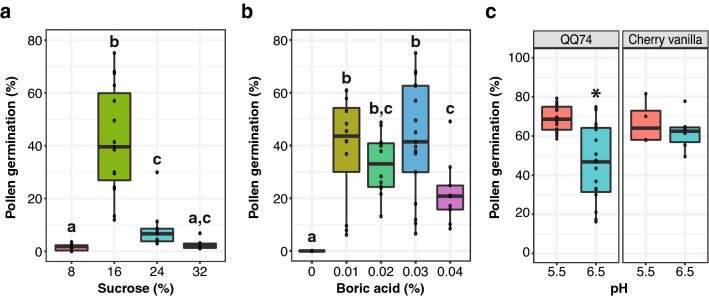
**a** Pollen germination percentage in response to sucrose concentration (%) in the germination medium in quinoa QQ74. Sample sizes are n = 9 for 8% sucrose medium, n = 15 for 16% sucrose medium, n = 9 for 24% sucrose medium, and n = 8 for 32% sucrose medium. **b** Pollen germination percentage in response to boric acid (%) in the germination medium in quinoa QQ74. Sample sizes are n = 10 for 0% boric acid, n = 12 for 0.01% boric acid, n = 12 for 0.02% boric acid, n = 17 for 0.03% boric acid, and n = 9 for 0.04% boric acid. **c** Pollen germination percentage in response to pH 5.5 and 6.5 in the germination medium in quinoa QQ74 and cherry vanilla. Sample sizes are n = 15 for QQ74 in pH 5.5 medium, n = 19 for QQ74 in pH 6.5 medium, n = 4 for cherry vanilla in pH 5.5 medium, and n = 6 for cherry vanilla in pH 6.5 medium. Letters or an asterisk above boxes indicate significant differences from Welch's t-test (p-value < 0.05)

Sucrose concentrations of 8%, 16%, 24%, and 32% (w/v) were evaluated for the reference genome quinoa accession QQ74 (PI 614886) to determine the optimal concentration of sucrose for in vitro pollen germination (Table 1). The highest germination was obtained with 16% sucrose with a median germination of 39.6%, which was significantly higher than 24% sucrose with a median germination of 6.6% (t-test p-value 1.7 × 10^–5^, Fig. 2a). An increase to 32% or a decrease to 8% of sucrose in the medium results in lower germination with a median germination of 2.2% and 1.8% respectively (Fig. 2a).

**Table 1:** Media used for sucrose concentration test

	Sucrose medium 1	Sucrose medium 2	Sucrose medium 3	Sucrose medium 4
Sucrose	8%	16%	24%	32%
Boric acid	0.01%	0.01%	0.01%	0.01%
Calcium nitrate	0.007%	0.007%	0.007%	0.007%
pH	5.5	5.5	5.5	5.5
Median germination	1.8%^a^	39.6%	6.6%^a^	2.2%^a^

^a^Indicates it was significantly different from the highest germination rate obtained (t-test p-value < 0.05)

### Optimization of boric acid concentration in pollen germination media

Boric acid concentrations of 0%, 0.01%, 0.02%, 0.03%, and 0.04% were evaluated for the reference genome quinoa accession QQ74 (PI 614886) to identify the optimal concentration of boric acid in the germination medium (Table 2). No boric acid in the medium resulted in no pollen germination, indicating that boric acid is essential for quinoa pollen germination (Fig. 2b). There were no significant differences in germination using 0.01%, 0.02%, or 0.03% boric acid in the medium (Fig. 2b). At 0.04% boric acid in the medium the germination was reduced significantly to 20.8% from 41.4% median germination obtained at 0.03% boric acid (t-test p-value 0.006) and from 43.6% median germination obtained at 0.01% boric acid (t-test p-value 0.029, Fig. 2b). There were no significant differences in germination comparing 0.02% to 0.04% boric acid (t-test p-value 0.06223). Therefore, boric acid at either 0.01% or 0.03% could be used for optimal germination. For this study, 0.03% boric acid was used in subsequent experiments.

**Table 2:** Media used for boric acid concentration test

	Boron medium 0	Boron medium 1	Boron medium 2	Boron medium 3	Boron medium 4
Sucrose	16%	16%	16%	16%	16%
Boric acid	0	0.01%	0.02%	0.03%	0.04%
Calcium nitrate	0.007%	0.007%	0.007%	0.007%	0.007%
pH	5.5	5.5	5.5	5.5	5.5
Median germination	0%^a^	43.6%	33%	41.4%	20.8%^a^

^a^Indicates it was significantly different from the highest germination rate obtained (t-test p-value < 0.05)

### Optimization of pollen germination media pH

Two pH levels (5.5 and 6.5) were evaluated in QQ74 (PI 614886) and cherry vanilla accessions (Table 3). Cherry vanilla was added to this experiment to test the efficiency of the germination medium in a different genotype. In QQ74 the highest germination was obtained at a pH of 5.5 with a median germination of 68.6% compared to 46.8% at a pH of 6.5 (t-test p-value 0.000107, Fig. 2c). Pollen germination in cherry vanilla has no significant differences between both pHs evaluated (t-test p-value 0.506), at pH 5.5 the median germination was 64.1% and 62.4% at pH 6.5 (Fig. 2c). Since for QQ74 germination at pH 5.5 was significantly higher than at pH 6.5, pH 5.5 was selected.

**Table 3:** Media used for pH test

	pH medium 1	pH medium 2
Sucrose	16%	16%
Boric acid	0.03%	0.03%
Calcium nitrate	0.007%	0.007%
pH	5.5	6.5
Median germination for QQ74	68.6%	46.8%^a^
Median germination for cherry vanilla	64.1%	62.4%

^a^Indicates it was significantly different from the highest germination rate obtained for that genotype (t-test p-value < 0.05)

### 48 hours of incubation was selected to evaluate pollen germination

**Fig. 3:**
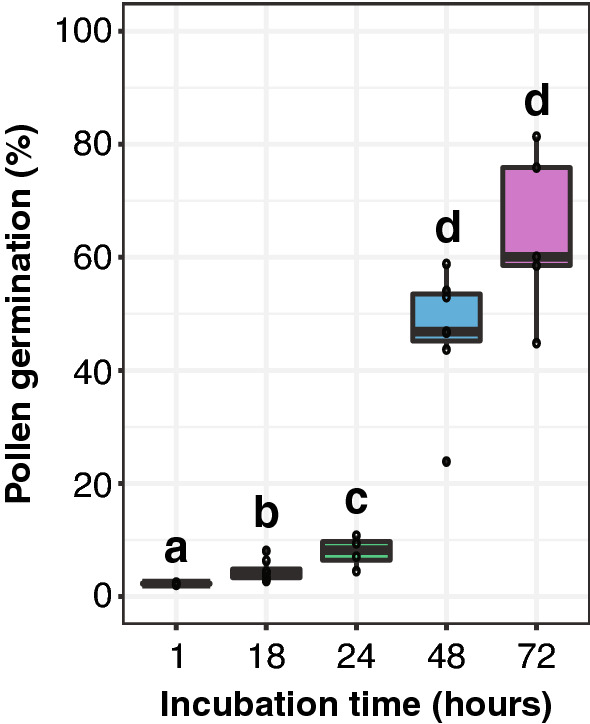
Pollen germination percentage in response to incubation time (hours) in quinoa QQ74. Sample sizes are n = 2 for 1 h of incubation, n = 8 for 18 h of incubation, n = 4 for 24 h of incubation, n = 7 for 48 h of incubation, and n = 5 for 72 h of incubation. Letters above boxes indicate significant differences from Welch's t-test (p-value < 0.05)

To determine the incubation time required for maximum germination under in vitro conditions, pollen germination was evaluated after 1, 18, 24, 48, and 72 h. Pollen germination was evaluated in a germination medium containing 16% sucrose, 0.03% boric acid, and 0.007% calcium nitrate at pH 5.5. There is a significant increase in germination up to 48 h of pollen incubation (Fig. 3). Pollen germination increased significantly from 8.2% median germination at 24 h to 46.9% median germination at 48 h of incubation (t-test p-value 0.00005, Fig. 3). Increasing the incubation time to 72 h did not result in significantly higher germination (t-test p-value 0.05953; Fig. 3), and resulted in visible contamination of the germination media, leading to sample loss. Therefore 48 h of incubation was selected.

### Timing of pollen collection is critical for successful pollen germination

**Fig. 4:**
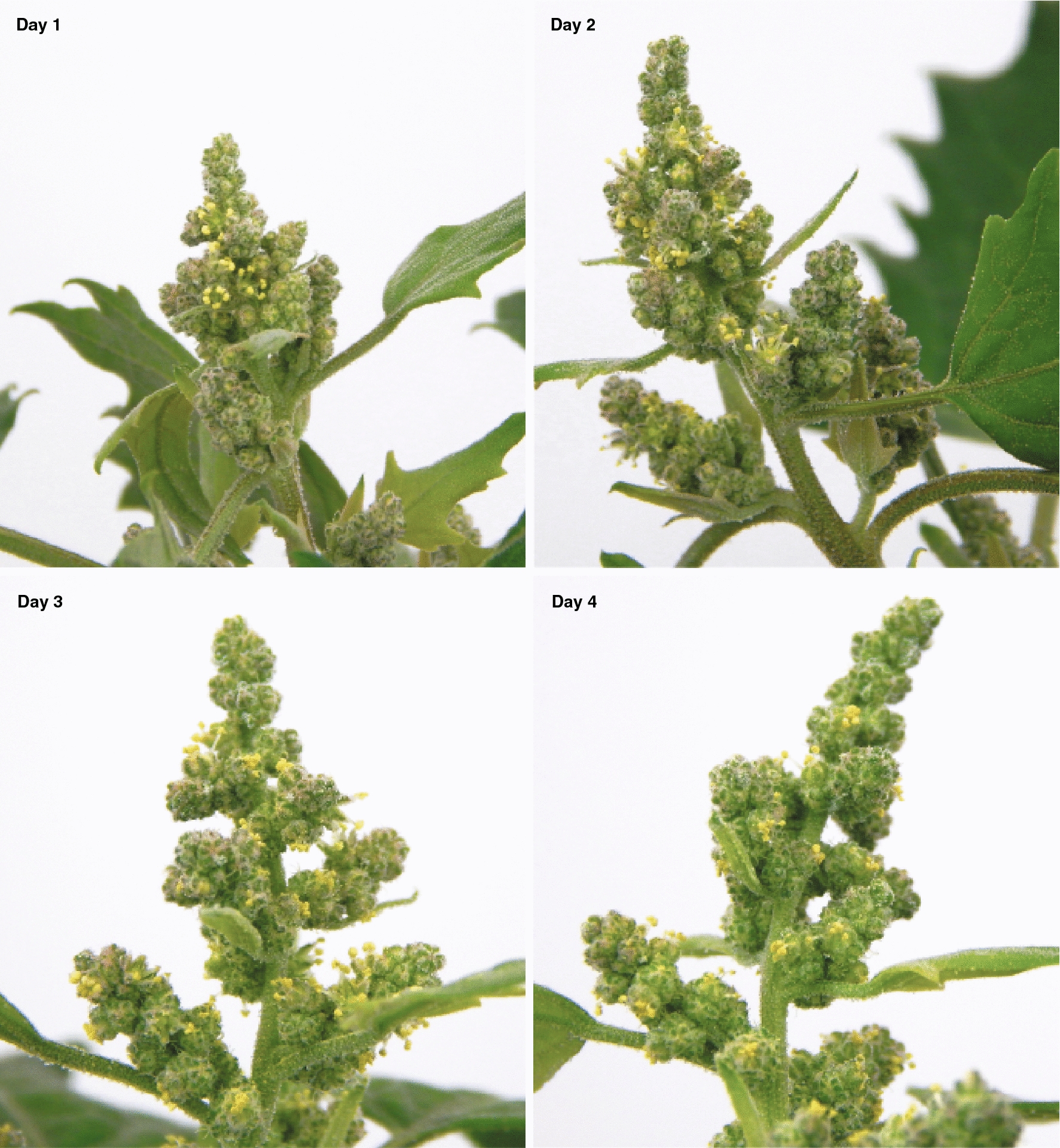
Panicle development after first anthesis. Images from a main panicle were taken at 1, 2, 3 and 4 days after first anthesis in quinoa QQ74

The flowering stage of quinoa panicles is critical for the success of the pollen germination assay. This is because quinoa produces many new flowers daily for several weeks during panicle development, and pollen viability depends on the development of each individual flower. Therefore, collecting pollen too early or too late during the flower development has a significant impact on pollen germination. First anthesis is defined as the time point when flowers open with extruded anthers for the first time in the plant. Starting at first anthesis, the number of hermaphrodite flowers with extruded anthers changes daily (Fig. 4). In the main panicle the first few flowers open then several new flowers keep opening daily for approximately one week. After this short period of rapid development there will be only a few or no new flowers opening daily for a few more weeks.

**Fig. 5:**
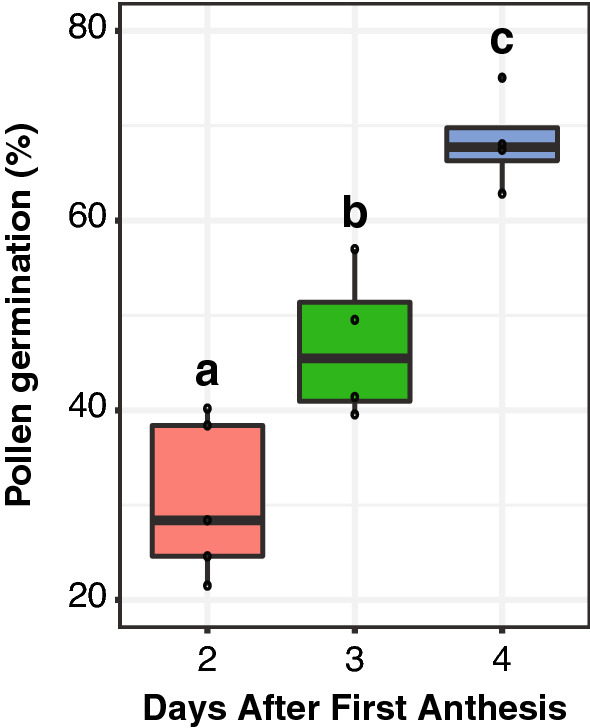
Pollen germination percentage in response to the time of pollen collection, measured as the days after first anthesis in quinoa QQ74. Sample sizes are n = 5 for 2 days AFA, n = 4 for 3 days AFA, and n = 4 for 4 days AFA

Pollen collected 2 to 4 days after the first anthesis (AFA) was evaluated to determine the best time for pollen collection. Pollen from day 1 AFA was not collected because few flowers per panicle were open and the anthers were not dehiscent. Pollen from day 5 AFA was not evaluated because by that time a significant amount of pollen from the main panicle had already fallen out of the flowers, making it very difficult to collect. Pollen germination was evaluated after 48 h of incubation in a germination medium containing 16% sucrose, 0.03% boric acid, and 0.007% calcium nitrate at pH 5.5. The highest germination was obtained from pollen collected at day 4 AFA with a median germination of 67.8% (Fig. 5). This was significantly higher than the 45.5% median germination obtained from pollen collected at 3 days AFA (t-test p-value 0.006) and the 28.4% median germination obtained from pollen collected at 2 days AFA (t-test p-value 0.00009, Fig. 5).

## Discussion

The quinoa pollen germination medium developed here showed an efficiency (~ 65%) comparable to media used to germinate pollen from grapes [22], wheat [13], and cotton [23], but slightly lower than the germination efficiency obtained in some of the most commonly studied plant species such as Arabidopsis (~ 90%) [24] and soybeans (~ 90%) [25]. This could indicate that future studies on pollen germination in other quinoa accessions and testing of other medium components may still lead to higher pollen germination efficiencies in quinoa.

The optimal sucrose concentration found in this study (16%) is slightly higher than that used for most pollen germination media (~ 10%) [26]. This may be because quinoa pollen is reported to be trinucleate [9]. Trinucleate pollen typically requires slightly higher sucrose for germination than binucleate pollen [26], which is more common in other species. For boric acid, we found two optimal concentrations: 0.01% and 0.03%. The concentration of 0.01% is equivalent to 1.6 mM boric acid, the median concentration used for most pollen germination media [26]. Although 0.03% boric acid is substantially higher than 0.01%, both concentrations yielded very similar germination efficiencies, indicating that quinoa may tolerate higher boric acid concentrations in pollen germination media than other species. However, from previous studies there is no indication that tolerance to a higher boric acid concentration in the germination media correlates to any particular pollen characteristics [26]. The optimal pH for quinoa accession QQ74 was 5.5, which is typically used for germination of starchy pollen [26]. This indicates that quinoa pollen may have higher starch content than most other species, which is typical of trinucleate pollen [26]. However, to our knowledge, the starch content of quinoa pollen has not been reported.

It is interesting that the optimal incubation time for quinoa pollen germination was 48 h. Reports in other species indicate that incubation periods longer than 24 h typically do not significantly increase germination efficiency [27–29]. This may indicate that quinoa pollen may be particularly resilient to loss of fertility over time after anther dehiscence.

A common problem for in vitro pollen germination is establishing a reproducible method [13, 30]. Working with quinoa pollen is particularly challenging, because quinoa can have many panicles in each plant, all at different developmental stages, and the flowers in each panicle also are at different developmental stages. Further, most quinoa flowers are female only, but pollen must be collected from the hermaphrodite flowers, which can be spread along the panicle. Therefore, pollen collection must be done carefully, tapping the panicle at different angles and from different parts to collect viable pollen. We showed that the key step for a successful in vitro pollen germination experiment is the developmental time point when quinoa pollen is collected. It is difficult for even an experienced researcher to visually detect the optimal time for pollen collection. Because of this, we measured overall panicle development using days after first anthesis (AFA). We showed that at 4 days AFA pollen germination peaked at 68.4% on average. This approach was likely successful in part because during early panicle development, flower development is much more uniform along the panicle than at later days AFA. At later time points there is the technical challenge that most pollen has fallen out of the flowers in the main panicle and it is likely that too much senescent and dry pollen would be collected, which has much lower germination rates. Although tracking the individual age of each hermaphrodite flower would likely yield a more accurate estimation of the age at which the pollen is most viable, such a study is technically unfeasible in quinoa. This is both because an individual flower yields a very small amount of pollen and because tracking quinoa’s very small flowers over time is extremely difficult. Overall, we showed that pollen collected from a relatively early time point in panicle development ensures more uniformly developed pollen, and a more accurate representation of peak pollen viability.

## Conclusions

We report for the first time an in vitro pollen germination protocol for quinoa, which we tested on two genome sequenced quinoa accessions: PI 614886 and cherry vanilla from different geographical origins. This method is a valuable tool to study pollen viability in quinoa. Pollen viability is known to be affected under temperature stress in many plant species, but it is unclear what happens in quinoa [8, 9]. This pollen germination method will help understand pollen viability in quinoa under stress. This pollen germination method could also be used to identify male-sterile quinoa lines for breeding. A tool to semi-automate the process of counting germinating pollen was also developed in the open-source image analysis toolkit, PlantCV.

Tracking days after the first anthesis was an effective way to estimate flower and pollen maturity during early flowering. This is important in quinoa because quinoa continuously produces new flowers during most of the period of panicle development, leading to significant differences in flower and pollen maturity among the many flowers within a panicle. Tracking days AFA is a relatively simple and reproducible way to collect optimal pollen for successful in vitro pollen germination studies.

## Materials and methods

### Plant material and pollen collection

*Chenopodium quinoa* seeds (accessions PI614886 and PI643079) were obtained from the USDA. Seeds were planted in Pro-Mix FPX (Premier Tech Horticulture, Quebec, Canada) soil and grown for 10 to 14 days at 22 °C, 12 h day photoperiod, 50% relative humidity, 400 µmol m^−2^ s^−1^ light intensity. Plantlets with four leaves were transplanted to Berger BM7 (Berger, Quebec, Canada) soil in 4.5-inch pots, and then transferred to a growth chamber set to the same conditions (22 °C, 12 h day photoperiod, 50% relative humidity, 400 µmol m^−2^ s^−1^ light intensity) until flowering. After the main inflorescence became visible, plants were observed daily to identify the first anthesis. First anthesis was defined as the first open flower with extruded anthers (Fig. 4). Plants were grouped according to the day of first anthesis to have similar maturity of flowers during pollen collection. Pollen was collected by tapping quinoa flowers from 3 to 5 plants into petri-dishes. Pollen from plants growing in growth chambers was collected 5 h after lights-on (zeitgeber time 5) and 2, 3, and 4 days after the first anthesis to determine the optimal day for maximum pollen germination. Immediately after pollen collection, pollen was added to germination media to avoid pollen desiccation.

### Optimization of germination medium

The culture medium developed by Hecker & McClintock, 1988 [16] for pollen germination of sugar beet was used as the initial medium to develop the quinoa pollen germination medium. To optimize the germination medium varying concentrations of sucrose and boric acid were evaluated. Calcium nitrate was kept at a constant concentration in all test media: 0.007% (w/v). Sucrose concentrations of 8%, 16%, 24%, and 32% (w/v) were first evaluated. After an optimum sucrose concentration was identified, boric acid concentrations were evaluated at 0%, 0.01%, 0.02%, and 0.03% (w/v). The culture medium was evaluated at pH 5.5 and 6.5. Media was filter sterilized with a 0.22 µm pore size nylon filter.

### Pollen tube germination conditions

**Fig. 6:**
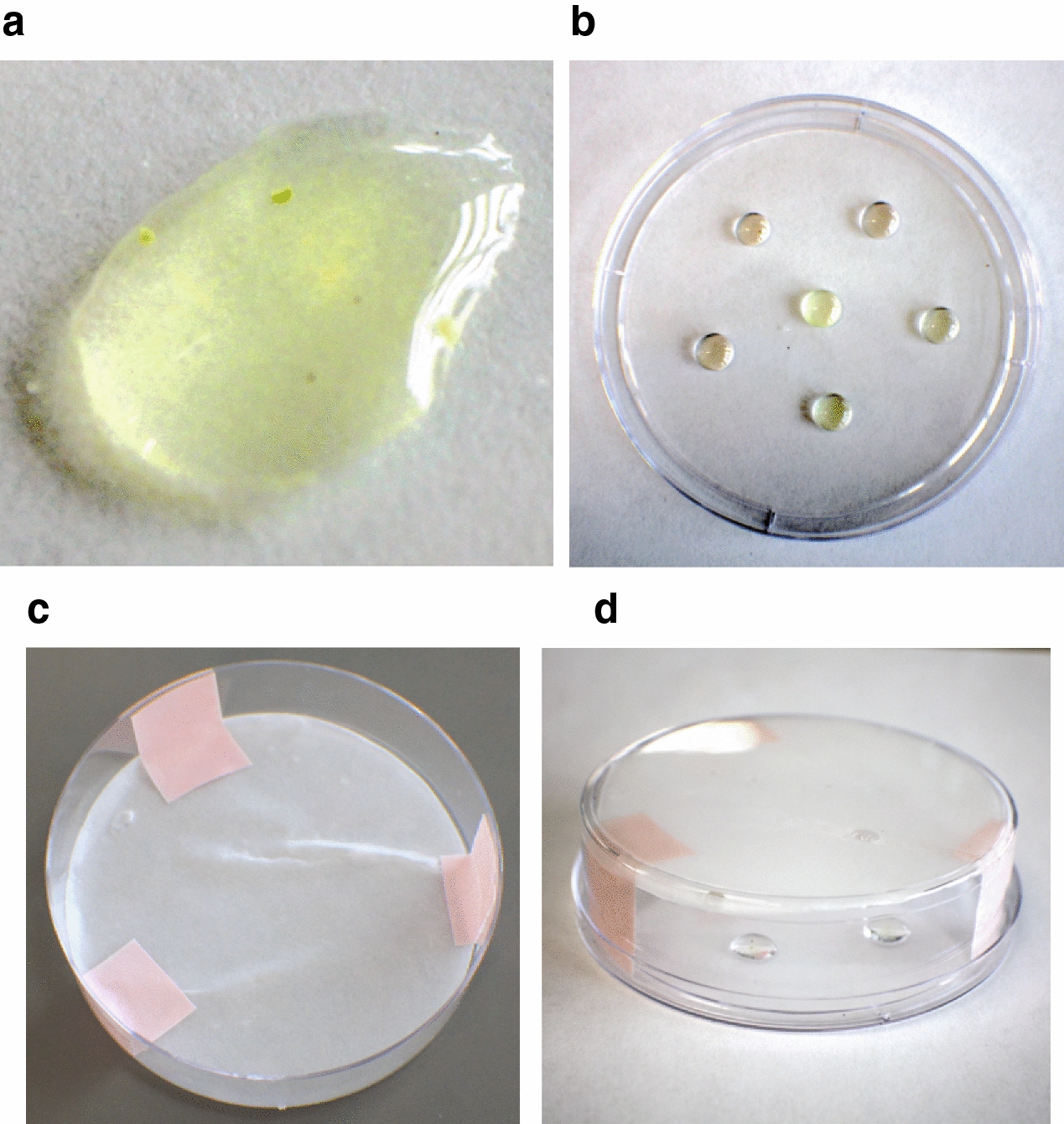
**a** Close-up of a germination medium droplet with the ideal concentration of pollen for maximum germination. **b** Petri dish lid with germination medium droplets containing pollen. **c** Petri dish bottom with wet filter paper taped on 3 points. **d** Incubation setup for pollen germination, before sealing with Parafilm

Pollen germination media was added to pollen in a sterile flow hood to minimize contamination of the plates during incubation. To incubate the pollen and allow germination, a humidity chamber was used following the procedure described by Ordoñez [31]. A sterile 1000 µl micropipette tip was used to gently mix pollen and pollen germination media. A concentration of approximately 75 µl of pollen germination medium/2.73 mg of pollen was used (Fig. 6a). This high density is important to obtain good germination [30]. The pollen and medium mix was then transferred to a deep petri dish lid (100 × 25 mm) as four to six droplets (~ 75 µl for each droplet, Fig. 6b). To maintain humidity inside the petri-dishes, each petri-dish had a sterile filter paper (Whatman grade 1 qualitative cellulose filter paper, Cytiva, Buckinhamshire, United Kingdom) wet with sterile distilled water (~ 2500 µl) placed in the bottom of the deep petri dish. The filter paper was taped in 3 points to the bottom of the petri-dish to hold it in place during the incubation (Fig. 6c). Any air pockets trapped between the petri dish and the moistened filter paper were pushed out with a sterile micropipette tip or a sterile loop. The petri-dishes were placed upside down (Fig. 6d) and sealed with Parafilm for incubation. Pollen was incubated for 48 h at 22 °C in a growth chamber with 12 h day photoperiod, 50% relative humidity, 400 µmol m^2^/s light intensity. Each droplet was considered a sample for data analysis.

### Imaging of pollen

Pollen germination was evaluated after 48 h of incubation. Samples were pipetted onto microscope slides using micropipette tips (1000 µl) that were clipped to make larger tip openings to minimize damage to the pollen tails. Pollen germination was observed using a Leica CTR6000 microscope, using 5X magnification. Images of approximately ten fields of view with an average of 50 pollen grains per view were captured per sample. Images are available here: https://doi.org/10.5281/zenodo.5909573.

Pollen can aggregate on the germination medium, making counting germinated and non-germinated pollen grains difficult. Pollen tubes can form a net and have many air sacs which help them float [32, 33], so they can aggregate at the top and center of the germination medium drop, while ungerminated pollen sinks. Since this can form a germination gradient in the drop, it is important to be consistent in the regions of the drop that are counted to calculate pollen germination. Pollen towards the center of the drop may have significantly higher germination rates, but will also overlap more frequently, making it harder to accurately count at the center of the drop. Counting pollen at the edges of the drop will be biased towards low germination rates. We counted pollen only in areas where the pollen was well spread over the microscopy slide with little or no overlap of the pollen grains (Fig. 1a), while avoiding the edges of the drop or areas where the rate of pollen germination was evidently very different from the rest of the drop.

### Semi-automated pollen counting tool in plantCV

PlantCV is a Python-based, open-source image analysis toolkit [20, 21]. A PlantCV image analysis workflow and new PlantCV modules were developed to semi-automate the process of detecting pollen grains and pollen tubes. The workflow consists of two main steps, first segmenting the objects (pollen grains and tubes) from the background, and then identifying only the objects that are likely to be pollen grains. After these two automatic steps, the user manually corrects incorrect detections and selects missing grains.

Segmentation of target objects (pollen grains or pollen tubes) from the background using the PlantCV function ‘*plantcv.threshold.binary’* in the blue channel of the RGB image with a threshold of 150. To clean up the segmentation we used the morphological operations ‘*plantcv.fill_holes’* and ‘*plantcv.fill’.*

In order to detect the pollen grains, we measured the eccentricity of each connected region in the segmentation. Circular objects have values of eccentricity close to 0. Thus, we identified pollen grains in the images by detecting any object with eccentricity under a value of 0.65. This was done using the new PlantCV function ‘*plantcv.detect_discs’.*

We then imported the results in the new PlantCV tool ‘*plantcv.visualize.ClickCount’* where we manually refined the detections. We used this same tool to manually identify the germinated grains. A pollen grain was counted as germinated only when the length of the tube was larger than the diameter of the grain. In the end we obtain the total number of grains and the number of germinated grains. From these measurements we can determine the number of non-germinated grains. This workflow is available at https://github.com/danforthcenter/quinoa-pollen-germination and a tutorial is availabe at https://github.com/danforthcenter/plantcv-tutorial-interactive-pollent-count**.**

### Determination of pollen germination rates

The pollen germination percentage was calculated for each image by multiplying the number of germinated pollen grains by 100 and dividing by the total number of pollen grains in the image. Then, the mean germination percent was calculated for each sample.

### Analysis and statistics

Data was analyzed using R version 4.1.2 and RStudio version 2021.09.1. A Welch’s t-test was used to establish statistically significant differences in germination rates between days after first anthesis, incubation periods, sucrose concentrations, pHs and boric acid concentrations.

### Pollen germination protocol for the lab

A laboratory protocol for germinating quinoa pollen can be found in Additional file 1: Pollen germination protocol for the lab.

## Supplementary Information

**Additional file 1: **Pollen germination protocol for the lab

## Data Availability

The image datasets generated and analyzed during the current study are available on Zenodo: https://doi.org/10.5281/zenodo.5909573. Scripts and extracted numerical data are available on GitHub: https://github.com/danforthcenter/quinoa-pollen-germination.
