# Supplementary material for: A protocol for Chenopodium quinoa pollen germination
Source: Plant Methods. 2022 May 18;18:65. doi: 10.1186/s13007-022-00900-3 (PMC9118578; doi:10.1186/s13007-022-00900-3)
Supplement: Supplementary file 1 — Additional file 1: Pollen germination protocol for the lab [file 13007_2022_900_MOESM1_ESM.pdf]

Pollen germination protocol for the lab

1. Pollen germination medium preparation: To make 100 ml of medium, take 70 ml of distilled water and add 16 g of sucrose, 3 ml from a stock solution of 0.01 g boric acid/ml, 1 ml of a stock solution of 0.01 g calcium nitrate tetrahydrate/ml (see table below). Mix the solution. Add water to the final volume of 100 ml and mix the solution again. The resulting solution will have a concentration of 16% sucrose, 0.03% boric acid, 0.007% calcium nitrate and will typically have a pH very close to 5.5. If needed, carefully adjust the pH to 5.5 with highly diluted solutions of sodium chloride or sodium hydroxide. Sterilize medium by filtration (e.g. 0.22 µm pore size nylon filter). Do not autoclave. Medium can be stored at 4°C for 1 week.

Medium component	Amount	Final concentration
Sucrose	16 g	16%
Boric acid stock solution (0.01 g/ml)	3 ml	0.03%
Calcium nitrate tetrahydrate stock solution (0.01 g/ml)	1 ml	0.007%
pH		5.5

2. In a flow hood working under sterile conditions prepare a humidity chamber (see image below) to incubate pollen. Using a deep petri-dish, tape a sterile filter paper disk to a petri-dish bottom (see image below) and wet the paper with a few drops of sterile distilled water. It will maintain humidity in the petri-dish during pollen incubation.

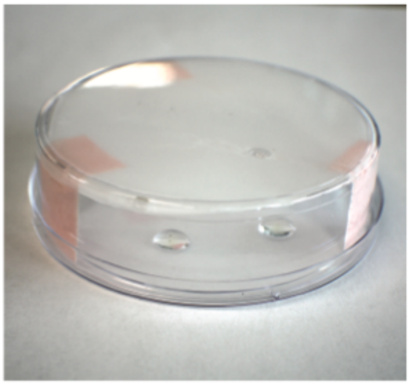

Humidity chamber

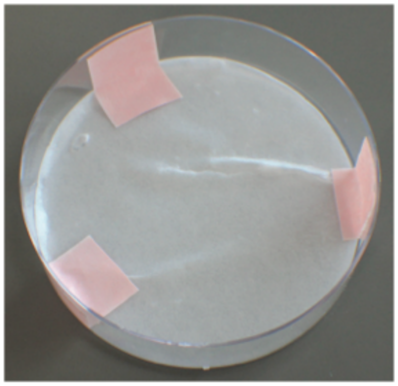

Petri dish bottom with filter paper

3. Collect pollen from the main panicles by tapping quinoa flowers into petri-dishes, we recommend for quinoa pollen to collect around 5 hours after lights-on (zeitgeber time 5). Pollen should be collected from plants 4 days after the first anthesis to obtain maximum germination.

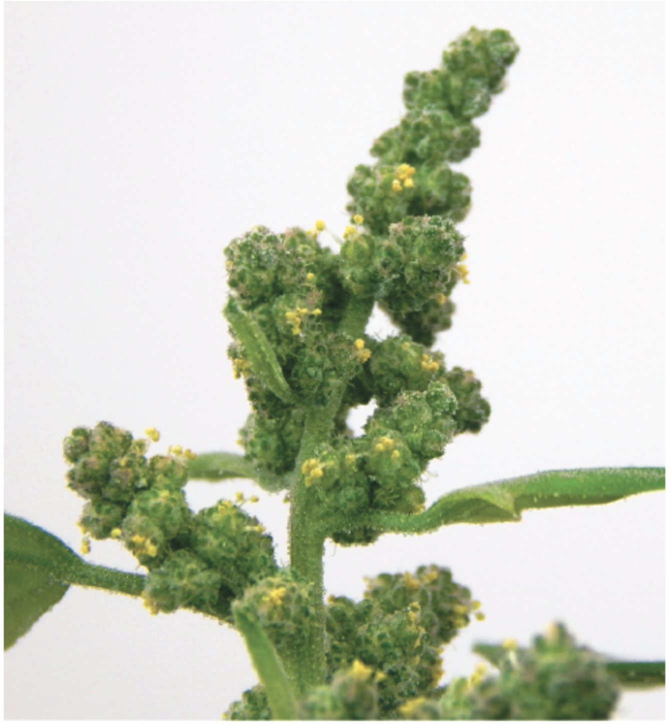

QQ74 main panicle at 4 days after first anthesis

4. As soon as pollen is collected, incubate it in the pollen germination medium. Add small drops of germination medium over the collected pollen to a final pollen concentration of approximately 2.73 mg for every 75 µl of medium in a petri dish (see image below) and then transfer ~ 75 µl (pollen+medium) drops to the lid of the humidity chamber prepared in step 1 (see image below). Close the humidity chamber and seal with parafilm, being careful so that the drops stay in place.

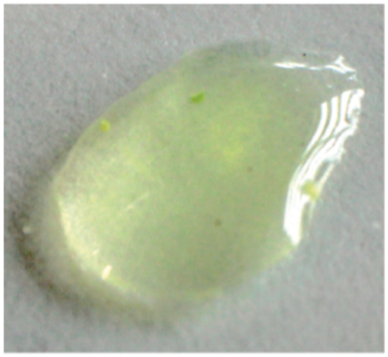

Pollen mixed with germination medium

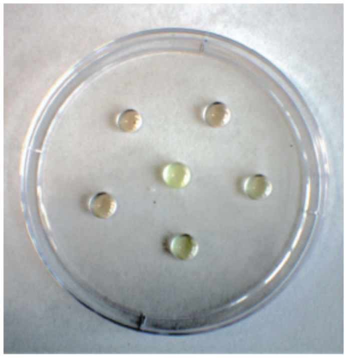

Drops of germination medium and pollen mixture

5. Incubate pollen at 22°C, 12 hour day photoperiod, 50% relative humidity, 400 µmol m^2/s light intensity, for 48 hours to obtain maximum pollen germination. Prepare microscope slides with the incubated pollen and medium. Pipette a sample of the incubated pollen to a microscopy slide using a wide mouth tip to avoid damaging the long pollen tubes and put a cover slide. Observe pollen under a microscope in a bright light field at approximately 5X magnification, then acquire images for image analysis.

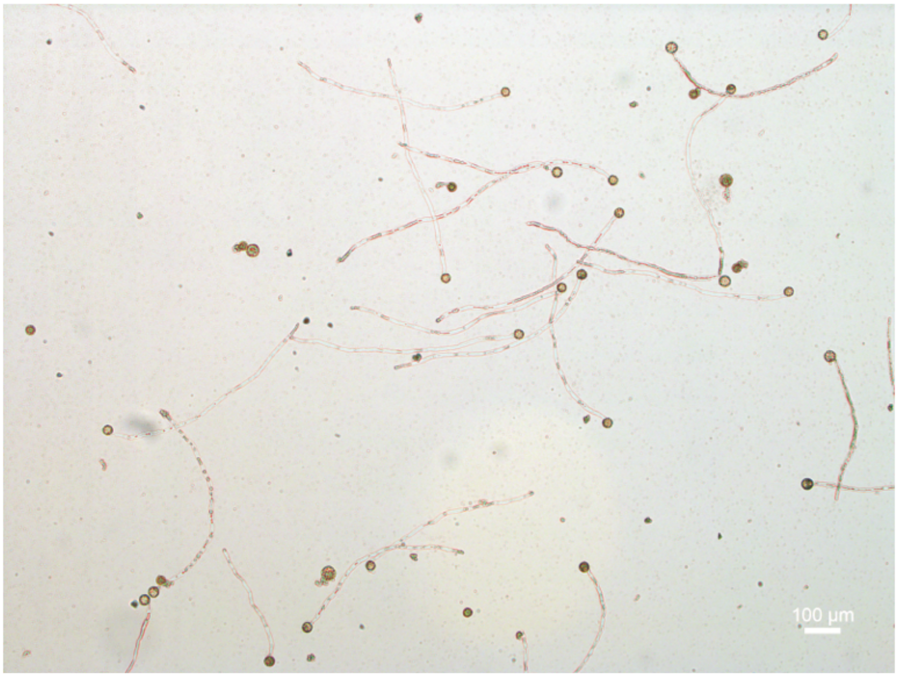

Example microscopy image of germinated pollen

6. Follow the tutorial at https://github.com/danforthcenter/plantcv-tutorial-interactive-pollent-count for image analysis, to obtain germinated, non-germinated, and total pollen counts.
